# The stress regulator FKBP51: a novel and promising druggable target for the treatment of persistent pain states across sexes

**DOI:** 10.1097/j.pain.0000000000001204

**Published:** 2018-03-12

**Authors:** Maria Maiarù, Oakley B. Morgan, Tianqi Mao, Michaela Breitsamer, Harry Bamber, Max Pöhlmann, Mathias V. Schmidt, Gerhard Winter, Felix Hausch, Sandrine M. Géranton

**Affiliations:** a Department of Cell and Developmental Biology, University College London, London, United Kingdom; b Department of Translational Research in Psychiatry, Max Planck Institute of Psychiatry, Munich, Germany. Drs. Tianqi Mao and Felix Hausch is now with Institute of Organic Chemistry and Biochemistry, Technical University Darmstadt, Darmstadt, Germany; c Department of Pharmacy, Pharmaceutical Technology and Biopharmaceutics, Ludwig-Maximilians-Universität München, Munich, Germany; d Department of Stress Neurobiology and Neurogenetics, Max Planck Institute of Psychiatry, Munich, Germany

**Keywords:** FKPB51, Dorsal horn, Glucocorticoid receptor, Glucocorticoid signalling, Persistent pain, Stress, Interleukin-6, Corticosterone, Pharmacological inhibition, Paclitaxel, Vesicular phospholipid gel

## Abstract

It is well established that FKBP51 regulates the stress system by modulating the sensitivity of the glucocorticoid receptor to stress hormones. Recently, we have demonstrated that FKBP51 also drives long-term inflammatory pain states in male mice by modulating glucocorticoid signalling at spinal cord level. Here, we explored the potential of FKBP51 as a new pharmacological target for the treatment of persistent pain across the sexes. First, we demonstrated that FKBP51 regulates long-term pain states of different aetiologies independently of sex. Deletion of *FKBP51* reduced the mechanical hypersensitivity seen in joint inflammatory and neuropathic pain states in female and male mice. Furthermore, *FKBP51* deletion also reduced the hypersensitivity seen in a translational model of chemotherapy-induced pain. Interestingly, these 3 pain states were associated with changes in glucocorticoid signalling, as indicated by the increased expression, at spinal cord level, of the glucocorticoid receptor isoform associated with glucocorticoid resistance, GR_β_, and increased levels of plasma corticosterone. These pain states were also accompanied by an upregulation of interleukin-6 in the spinal cord. Crucially, we were able to pharmacologically reduce the severity of the mechanical hypersensitivity seen in these 3 models of persistent pain with the unique FKBP51 ligand SAFit2. When SAFit2 was combined with a state-of-the-art vesicular phospholipid gel formulation for slow release, a single injection of SAFit2 offered pain relief for at least 7 days. We therefore propose the pharmacological blockade of FKBP51 as a new approach for the treatment of persistent pain across sexes, likely in humans as well as rodents.

## Abstract 2

Supplemental Digital Content is Available in the Text.

Pharmacological blockade of FKBP51 can reduce established persistent pain states across sexes.

## 1. Introduction

The FK506 binding protein 51 (FKBP51) is important for the regulation of the stress response, and polymorphisms of the FKBP51-encoding gene, *FKBP5*, have been associated with atypical stress axis reactivity, with high levels of FKBP51 protein associated with a hyperreactive stress pathway often seen in patients with depression.^5,43^ Supporting these observations, deletion of *FKBP5* in mice leads to a reduction in corticosterone secretion and anxiety-related behaviours.^17,18^ FKBP51 is a co-chaperone that changes folding and activity of other proteins. It modulates the stress axis, also called the hypothalamic–pituitary–adrenal (HPA) axis, through binding of the steroid complex and inhibition of the glucocorticoid receptor (GR) function.^43^ Although it remains unclear whether FKBP51 binding to the steroid complex directly inhibits GR function,^12^ by regulating GR signalling FKBP51 can modulate the actions of glucocorticoids, including the expression of glucocorticoid-responsive genes and the GR-mediated negative feedback of the HPA axis.^43^

Traumatic events are powerful stressors that can activate the HPA axis and thereby are likely to contribute to the development of persistent posttraumatic pain in vulnerable individuals.^13,23,29^ Supporting this hypothesis, variations in *FKBP5* associated with enhanced induction of *FKBP5* mRNA by cortisol and reduced GR sensitivity^4,5^ were associated with increased musculoskeletal pain after motor vehicle collision or sexual assault.^7^ Interestingly, the association between the reported pain and the *FKBP5* gene variations was stronger 6 weeks after trauma than immediately after trauma, suggesting that *FKBP5* was more likely to contribute to persistent than acute pain states.^7^

Consistent with these findings, we have recently demonstrated that FKBP51 is expressed in the rodent dorsal horn where it plays a key role in the full development and maintenance of chronic joint inflammatory pain,^15,21^ but has no effect on acute pain responses. *FKBP5* knock-out (KO) male mice with an inflamed joint had reduced pain compared with their wild-type (WT) littermates, and inhibition of FKBP51 at spinal level, using siRNA, significantly attenuated preexisting joint inflammatory pain. The expression of FKBP51 was significantly reduced in the dorsal horn by intrathecal delivery of the siRNA but left intact in dorsal root ganglia, indicating that FKBP51 expressed in dorsal root ganglia was unlikely to contribute to the maintenance of the pain state. Moreover, FKBP51 modulation of long-lasting mechanical hypersensitivity occurred through the regulation of GR signalling,^21^ a signalling pathway previously shown to regulate the hypersensitivity seen in long-term pain states at spinal cord level.^33,39,40^ Finally, the deletion of FKBP51 was associated with a reduced expression at spinal cord level of the cytokine interleukin-6 (IL6), which is upregulated in chronic pain states^44^ and under transcriptional control of GR.^27,38^

Here, we have used molecular and pharmacological tools to investigate the regulation of permanent pain states of different aetiology by FKBP51, in both male and female mice. It has been suggested that the mechanisms of development of chronic pain states differed between sexes, implying a potential need for different strategies to address chronic pain in men and women.^2,32^ It was therefore crucial to investigate whether FKBP51 drove permanent pain states across sexes.

## 2. Material and methods

### 2.1. Animals

Subjects in all experiments were adult male and female mice (8-12 weeks old). Mice were FKBP51 KO and their WT littermates obtained from FKBP51 heterozygous from C.A. Dickey's group (University of South Florida). These mice were from mixed genetic background, C57Bl/6J and Swiss Webster. All animals were kept in their home cages in a temperature-controlled (20 ± 1°C) environment, with a light–dark cycle of 12 hours (light on at 7:30 am); food and water were provided ad libitum. All efforts were made to minimise animal suffering and to reduce the number of animal used (UK Animal Act, 1986).

### 2.2. Genotyping

For genotyping, DNA was extracted from a small portion of ear tissue. Each sample was lysed in 75 μL of alkaline lysis buffer (25 mM NaOH, 0.2 mM EDTA, pH 12) and heated at 95°C for 30 minutes. After cooling, 75 μL of neutralising reagent (40 mM Tris-HCl, pH 5) was added to each sample. The following primers were used for the polymerase chain reaction (PCR): forward primer (for WT and 51KO): AAAGGACAATGACTACTGATGAGG; reverse WT primer: AAGGAGGGGTTCTTTTGAGG; and reverse 51KO primer: GTTGCACCACAGATGAAACG. Amplification was obtained starting from 1 μL of DNA and 1 unit of Taq DNA polymerase (Promega, Madison, WI) in a final volume of 20 μL of amplification buffer. Fifteen microliter of products obtained by amplification was loaded on a 2% agarose gel containing 20 μL ethidium bromide, subjected to electrophoresis, and visualized under a UV light. Samples from WT animals showed a single PCR product of 363bp; samples from KO animals showed a single PCR product of 510bp; and samples from HET animals presented both bands.

### 2.3. Pain models

#### 2.3.1. Complete Freud adjuvant–induced ankle joint inflammation

Inflammation was induced by injection of complete Freud adjuvant (CFA; Sigma, United Kingdom) at the volume of 5 μL, in the left ankle joint, under isoflurane anaesthesia induced in a chamber delivering 2% isoflurane combined with 100% O_2_ and maintained during injection using a face mask. The needle entered the ankle joint from the anterior and lateral posterior position, with the ankle kept in plantar flexion to open the joint. Sham treatment consisted of anesthetizing the animals.

#### 2.3.2. Neuropathic model: spared nerve injury

The spared nerve injury (SNI) was performed as previously described.^11,21^ Briefly, under isoflurane anaesthesia, the skin on the lateral surface of the thigh was incised and a section made directly through the biceps femoris muscle exposing the sciatic nerve and its 3 terminal branches: the sural, the common peroneal, and the tibial nerves. The common peroneal and the tibial nerves were tight ligated with 5-0 silk and sectioned distal to the ligation. Great care was taken to avoid any contact with the spared sural nerve. Complete haemostasis was confirmed and the wound was saturated. Sham treatment consisted of exposing the sciatic nerve only.

#### 2.3.3. Chemotherapeutic-induced neuropathic pain: paclitaxel

To induce mechanical hypersensitivity, paclitaxel was injected under isoflurane anaesthesia as previously described.^26^ Paclitaxel (Sigma) was dissolved in a solution made up of 50% Cremophor EL (Sigma) and 50% absolute ethanol and stored at −20°C, for a maximum of 14 days, and was diluted in normal saline (NaCl 0.9%), just before administration. The vehicle for paclitaxel, 50% Cremophor EL and 50% absolute ethanol, was diluted, at the time of injection, with saline. Paclitaxel (2 mg/kg) was injected intraperitoneally (i.p.), on alternating successive days (days 0, 2, 4, and 6). No weight loss or mortality was observed in paclitaxel-treated mice throughout the experiments.

### 2.4. Behavioural testing

N.B: the experimenter was always blind to genotype and treatment.

#### 2.4.1. Von Frey's hairs

Animals were placed in Plexiglas chambers, located on an elevated wire grid, and allowed to habituate for at least 1 hour. After this time, the plantar surface of the paw was stimulated with a series of ascending forces Von Frey's monofilaments. The threshold was determined using the up-down method as described by Chaplan et al.^10^ The data were expressed as log of the mean of the 50% pain threshold ± SEM.

### 2.5. Intrathecal injections

The intrathecal injections were performed as previously described.^21^ Briefly, under anaesthesia, mice were held firmly, but gently, by the pelvic girdle using the thumb and forefinger of the nondominant hand. This grip caused the hind legs to splay outward and downward. The skin above the iliac crest was pulled tautly to create a horizontal plane where the needle was inserted. Using the other hand, the experimenter traced the spinal column of the mouse, rounding or curving the column slightly to open the invertebrate spaces. A 30-gauge needle connected to a 10 μL Hamilton syringe was used to enter between the vertebrae. After injection, the syringe was rotated and removed and posture and locomotion were checked. All intrathecally delivered drugs were injected in a 2 μL volume.

### 2.6. Drugs

Mifepristone (1 nmol in 2 μL) was purchased from Sigma and was dissolved in 10% ethanol in saline. Control animals received 2 μL of 10% ethanol in saline. SAFit2 was synthesized as previously described^14^ and used at a concentration of 2 mg/mL in vehicle A (4% ethanol, 5% PEG400, and 5% TWEEN 80 in 0.9% saline; used at 10 mL/kg or 20 mg/kg) or 10 mg/mL in vesicular phospholipid gel (VPG) = vehicle B (50% (m/m) egg lecithin containing at least 80% phosphatidylcholine in 10 mM phosphate-buffered saline (PBS) pH 7.4; used at 10 mL/kg or 100 mg/kg) or in vehicle C (20% EtOH, 40% PPG, 5% PEG400, and 5% TWEEN 80 in 0.9% saline; used at 10 mL/kg or 100 mg/kg). Vesicular phospholipid gels (with and without SAFit2) were prepared by a dual asymmetric centrifugation (DAC) technique as described below. Egg lecithin (Lipoid E80) was a kind gift from Lipoid GmbH (Ludwigshafen, Germany). Control animals received vehicle only.

#### 2.6.1. Preparation of vesicular phospholipid gels by dual asymmetric centrifugation

Vesicular phospholipid gels with a phospholipid amount of 50% (m/m) were prepared by DAC. To encapsulate the poorly water-soluble SAFit2 into the formulation by a direct incorporation method, the accurately weighted phospholipid was solved in ethanol (100%) and SAFit2 stock solution (20.0 mg/mL in 100% ethanol) was added in the desired amount to the phospholipid solution. The ethanol was evaporated for 2 days in a vacuum drying oven at a temperature of 25°C and a pressure of 10 mbar. Then, the solid mixture was hydrated with the accurate amount of 10 mM PBS buffer pH 7.4 and homogenized by a DAC (Speedmixer DAC 150.1 FVZ; Hauschild GmbH &Co KG, Hamm, Germany).

Dual asymmetric centrifugation is a double centrifugation technique which is perfectly suited for the mixing of highly viscous formulations and has been used in the past to prepare liposomes and fat emulsions.^19,35^ Its application to manufacture VPGs has been first described by Massing et al.^22^ in 2008. In this technique, the container is not only spun around the commonly known centrifugation axis in the middle of the centrifuge but also around a second axis in the middle of the container. Because of the 2 counterrotating movements, high shearing forces and a homogenous mixture of the components are achieved.

For homogenization of the phospholipids and buffer in our study, a process speed of 3500 rpm was used. A custom-made cooling system was installed to prevent the material from heating during the continuous mixing over 45 minutes. The final amount of SAFit2 in the formulation was 10 mg/g.

The described manufacture process was used for the preparation of a second VPG formulation without the addition of SAFit2 stock solution as a control formulation (vehicle B).

#### 2.6.2. Preparation of an SAFit2 solution with a concentration of 10 mg/mL

A nonretarded SAFit2 control solution was prepared to evaluate the depot effect of SAFit2-VPG. Therefore, vehicle C (20% ethanol, 40% propylene glycol, 5% polyethylene glycol 400%, and 5% Tween 80 in 0.9% saline) was needed to allow dissolving SAFit2 in a concentration of 10 mg/mL. Using this formulation, the direct comparison of the subcutaneous injection of the exact same dose and injection volume of SAFit2 solution and SAFit2-VPG was possible.

### 2.7. Blood sampling and corticosterone assay

Blood was collected in the morning between 9:00 and 11:00 from the mouse tail vein. Before sampling, mice were placed in a warming cabinet (39°C for 10-15 minutes) to dilate the blood vessel. The levels of corticosterone were measured using an ELISA kit (ab108821; Abcam, Cambridge, United Kingdom) following the manufacturer protocol.

### 2.8. Fresh tissue collection and RNA preparation

For fresh tissue collection, animals were terminally anesthetized with CO_2_ 3 days after CFA or sham surgery. The spinal cord segment corresponding to the lumbar area was rapidly removed, and the ipsilateral dorsal horn quadrants L4 to L6 were dissected out and frozen on dry ice. Samples were then stored at −80°C until further processing. Total RNA was extracted using an acid phenol extraction method (TRIzol reagent, RNeasy mini-columns; Quiagen, United Kingdom). RNA concentration was measured using the Nanodrop (Labtech International, Ringmer, United Kingdom).

### 2.9. RTqPCR

RNA samples were treated with DNase I (Quiagen, Crewley, United Kingdom). Equal amounts (between 200 and 500 ng depending on the set of experiments) of total RNA were reversed transcribed using random nonamers (Sigma, Poole, United Kingdom), Oligo (dT) 20 primers (Promega), and Superscript TM III RT (Invitrogen, Carlsband, CA) for 1 hour at 50°C in a total reaction volume of 20 μL. cDNAs were immediately used for qPCR or kept at −20°C; qPCR reactions were performed with DNA Engine (BioRad, Hercules, CA) using SYBR Green JumpStart Taq ReadyMix (Sigma, Poole, United Kingdom) and gene-specific primer sets (primer sequences available on request). One microliter of cDNA diluted 1/10 in H_2_O was amplified in a 3-step cycling program in a final reaction volume of 25 μL. Control cDNA samples obtained without transcriptase were always included, as well as samples without any cDNA template. Reactions were performed at least in triplicate and the specificity of the products was determined by melting curve analysis. The ratio of the relative expression of target genes to β-actin was calculated using the 2^ΔCt^ formula. Efficiencies of qPCRs were calculated for each gene using serial dilution.

### 2.10. Immunohistochemistry

For immunohistochemistry, mice were deeply anesthetized with pentobarbital and perfused transcardially with saline containing 5000 IU/mL heparin followed by 4% paraformaldehyde in 0.1 M phosphate buffer (PB; 25 mL per adult mouse). Lumbar spinal cords were dissected out, postfixed in the same paraformaldehyde solution for 2 hours, and transferred into a 30% sucrose solution in PB containing 0.01% azide at 4°C, for a minimum of 24 hours. Spinal cords were cut on a freezing microtome set at 40 μm.

For fluorescent immunohistochemistry, sections were left to incubate with primary antibodies O/N at room temperature (anti-FKBP51, 1:5000, Santa-Cruz #sc11518; anti-IL6, 1:5000, Abcam #ab6672). Appropriate biotinylated secondary antibody was used at the concentration of 1:400 and left for 90 minutes. Sections were then incubated with avidin biotin complex (ABC Elite, Vector Lab; 1:250 Vectastain A plus 1:250 Vectastain B) for 30 minutes followed by a signal amplification step with biotinylated tyramide solution (TSA; 1:75 for 7 minutes; Perkin Elmer, Wellesley, MA). Finally, sections were incubated with FITC avidin for 2 hours (1:600). For multiple labelling, other primary antibodies were left O/N at room temperature at the end of the TSA protocol (anti-Iba1 1:1500; Wako, Osaka, Japan; anti-GFAP 1:4000, DAKO, Cambridge, United Kingdom; anti-NeuN: 1:1000) and revealed the following day with appropriate direct secondary antibody at a concentration of 1:500 (Alexa Fluor). Finally, sections were incubated with the acid nucleic marker To-pro, as per manufacturer instructions. All fluorescent sections were coverslipped with Gel Mount Aqueous Mounting Medium (Sigma) to protect the fluorescence from fading and stored in dark boxes at +4°C.

For β-galactosidase and DAB staining, spinal cords were sectioned on a freezing microtome set to 20 μm. Free-floating sections were incubated in warmed X-gal working solution (X-gal stock; X-gal, Chem-Cruz #sc280488, in dimethylformamide; diluted in X-gal dilution buffer; 5 mM potassium ferricyanide crystalline, 5 mM potassium ferricyanide trihydrate, and 2 mM magnesium chloride in PB; 1:40) at 37°C for 1.5 hours. After the stain, the sections were washed in PB and then water for 5 minutes each. To achieve double labelling, 3,3′-diaminobenzidine (DAB) staining was used. First, the sections were blocked in PB with 3% serum, 3% triton, and 2% H2O2 for 1 hour, and then incubated in the chosen primary antibody either O/N (anti-NeuN, 1:1000, Millipore #MAB377; anti-NG2, 1:500, Millipore #MAB5384; anti-APC, 1:200, Calbiochem #OP80) or for 72 hours (anti-Iba1, 1:500; Wako). The sections were then incubated in appropriate secondary antibodies at 1:500 for 2 hours, followed by incubation with avidin biotin complex (ABC Elite, Vector Lab; 1:1000 Vectastain A plus 1:1000 Vectastain B) for 1 hour. The substrate was then developed using a peroxidase substrate DAB kit (Vector #SK4100) at optimised times, and the sections were washed and mounted. The following day, the sections were dehydrated in increasing ethanol concentrations (70%, 70%, 95%, 95%, 100%, 100%, histoclear ×2) and coverslipped with DPX. For the FKBP51 double DAB labelling, the sections underwent an initial DAB staining with a chosen primary antibody, as previously described. The sections were then incubated in anti-FKBP51 (1:500, Santa-Cruz #sc11518) for 72 hours and a second DAB staining procedure was completed, with the addition of a Nickel solution to the substrate mixture, to produce the distinguishable gray stain.

### 2.11. Image analysis

#### 2.11.1. Fluorescent images: confocal imaging and analysis

All fluorescent images of quadruple stain tissue were acquired by confocal microscopy using a laser-scanning microscope (Leica TCS NT SP). Sequential laser channel acquisition was used to prevent the generation of false positives by “bleed through” of immunofluorescence from 1 channel to the other. For quantitative analysis of cell-specific expression of FKBP51, single plan images were acquired with an ACS APO 40.0x oil objective (Figs. 2B and C). A single picture per section side was taken spanning laminae I to III. Offset, gain, and laser strength of each scan was not changed throughout the imaging. Five dorsal horn sections were imaged per animal for each ipsilateral and contralateral side, n = 3/group. Cell-specific expression was manually quantified from confocal images and individual optical plan inspection. For a cell (neurone, astrocyte, or microglia) to be considered positive for FKBP51, the expression of FKBP51 had to appear together with the cell-specific marker (or surrounded by the cell-specific marker for Gfap), and with the nucleic acid stain To-pro which labels all nuclei. We quantified the number of neurones, astrocytes, and microglia positive for FKBP51 and expressed it as a percentage of total number of neurones, astrocytes, or microglia.

#### 2.11.2. β-Galactosidase and DAB images

Images of β-galactosidase and DAB tissue were acquired using a Zeiss Axio Scan Z1 Slide Scanner bright field microscope. This would not allow the dissection of individual optical channels. Entire spinal cord sections were imaged using this system, with 5 to 6 dorsal horn sections imaged per animal, n = 3/group. Cell-specific expression was manually quantified from acquired images. For a cell (neurone or microglia) to be considered positive for FKBP51, X-gal stain had to appear within the cell-specific marker (NeuN or Iba1). We quantified the number of neurones and microglia positive for FKBP51 and expressed it as a percentage of total number of neurones or microglia.

### 2.12. Data analysis

All statistical tests were performed in IBM SPSS statistic 20. For the behavioural experiments, statistical analysis was performed on the data normalized by log transformation (Von Frey data; as suggested by Mills et al.^25^). The significance of any differences in sensitivity was assessed using repeated-measured 2-way or 1-way analysis of variance (ANOVA), as appropriate. In all cases, a significant effect of the main factor(s), or interactions between them, was taken as the criterion for progressing to post hoc analysis. Bonferroni analysis was the preferred post hoc analysis; however, if the general ANOVA was significant but no Bonferroni significance observed, we also reported the results of the least significant difference post hoc analysis. In all cases, “time” was treated as a within-subjects factor and “genotype” and “treatment” were treated as between-subject factors. For the RTqPCR experiments, data were analysed by univariate analysis for individual genes followed by the appropriate post hoc analysis (1-way ANOVA or *t* test). Biological samples for the measures of glucocorticoid levels were analysed by 2-way ANOVA and appropriate post hoc analysis.

### 2.13. Study approval

All procedures described in this article comply with the UK Animals (Scientific Procedures) Act 1986 and the research was performed under strict Home Office regulations, under the project licence PPL 70/7944.

## 3. Results

### 3.1. FKBP51 deletion significantly attenuates the development of mechanical hypersensitivity in long-term pain states across sexes

#### 3.1.1. Inflammatory pain states

**Figure 1.**
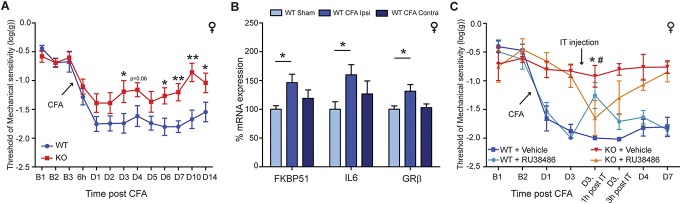
FKBP51 regulates long-term joint inflammatory pain states in female mice by modulation of glucocorticoid signalling. (A) Mechanical sensitivity in female knock-out (KO) and wild-type (WT) mice after injection of CFA in the ankle joint (n = 8/8) (2-way analysis of variance (ANOVA), TIME B3 to D14, factor “Genotype”: F_1,14_ = 11.3, *P* < 0.01). B: Baseline; D: day. Data show mean ± SEM. *P < 0.05, **P ≤ 0.01, WT vs KO; post hoc 1-way ANOVA. (B) RT-qPCR analysis of FKBP51, IL6, and GRβ in the dorsal horn 3 days after CFA injection in the ankle joint; data normalized to WT sham (for FKBP51 data, ANOVA: F_2,18_ = 3.53; *P* < 0.05; post hoc analysis least significant difference (LSD) *P* < 0.05. For GRβ data, ANOVA: F_2,18_ = 3.44; *P* < 0.05 post hoc analysis LSD *P* < 0.05. For IL6 data, ANOVA: F_2,18_ = 2.72; *P* < 0.1; post hoc analysis LSD *P* < 0.05). Data show mean ± SEM. *P < 0.05. (C) RU38486 (mifepristone, GR antagonist) effect on CFA-induced mechanical hypersensitivity in female WT mice and female KO mice (n = 4/7). Wild-type/vehicle vs WT/RU38486: 2-way ANOVA, 1 h-D4, factor “Treatment”: F_1,9_ = 5.4, *P* < 0.05; KO/vehicle vs KO/RU38486: 2-way ANOVA, TIME B2 to 1 hour, factor “Treatment”: F_3,18_ = 3.3, *P* < 0.05. B: Baseline; D: day. Data show mean ± SEM. *P < 0.05: WT/vehicle vs WT/RU38486; ^#^*P* < 0.05: KO/vehicle vs KO/RU38486; results of post hoc 1 way ANOVA. CFA, complete Freud adjuvant; GR, glucocorticoid receptor; IT, intrathecal.

We first examined whether FKBP51 regulated the development of the mechanical hypersensitivity seen with ankle joint inflammation in female mice through modulation of glucocorticoid signalling, as we have reported in male mice.^21^ We first tested the effect of global deletion of FKBP51 on the mechanical hypersensitivity that develops after the injection of CFA in the ankle joint. Global deletion of FKBP51 in female mice did not affect their basal mechanical threshold when compared with WT mice (Fig. 1A). However, after CFA injection in the ankle joint, although both WT and KO mice showed a significant decrease in mechanical sensitivity, the effects observed in KO mice were significantly less severe than in WT mice (Fig. 1A), as previously observed in males.^21^

Next, using RT-qPCR, we measured a significant increase in spinal FKBP51 mRNA in WT female mice after induction of joint inflammation with CFA (Fig. 1B), consistent with our previous findings.^21^ This increase was accompanied by an upregulation of IL6 and of the GR isoform associated with glucocorticoid resistance,^20^ GR_β_ (Fig. 1B), suggesting that glucocorticoid signalling was likely to contribute to the development of this long-term pain state in females, as seen in males.

Crucially, FKBP51 and IL6 were not upregulated in the dorsal horn of mice after IL6 injection in the hind paw (for FKBP51: sham 100 ± 7 vs IL6-treated ipsi: 120 ± 21; for IL6: sham 100 ± 14 vs IL6-treated ipsi: 149 ± 41; mean ± SEM, 24 hours post IL6, data normalised to WT; n = 6/6). Injection of IL6 in the hind paw induces short-term inflammation which is not modulated by FKBP51 (no differences in behaviour between FKBP51 KO and WT mice), but induces a mechanical hypersensitivity of a similar degree than that seen with ankle joint inflammation.^21^

Finally, we tested the hypothesis that glucocorticoid signalling contributes to the development of CFA-induced joint pain in female mice and investigated whether FKBP51 regulation of this pain state occurred through the modulation of glucocorticoid signalling. For this, we used the GR antagonist mifepristone (RU38486). When mifepristone was administered intrathecally 3 days after CFA injection in the ankle joint, there was a rapid reduction in the CFA-induced hypersensitivity in WT mice (Fig. 1C), indicating that GR signalling was crucial to the full manifestation of the hypersensitivity. However, we observed an increase in the hypersensitivity in *FKBP5* KO mice after mifepristone (Fig. 1C), indicating that, after *FKBP5* deletion, GR signalling in long-term pain states was antinociceptive. All together, these results were consistent with our findings in males and demonstrated that FKBP51 regulates inflammatory pain states in a sex-independent manner and, at least partly, by modulating GR signalling and possibly through the regulation of IL6 expression. Supporting this idea, we observed that IL6 was mainly expressed in neurones in the superficial dorsal horn of mice with an inflamed joint (Fig. S1, available online as supplemental digital content at http://links.lww.com/PAIN/A550), as we had previously reported for FKBP51.^21^

#### 3.1.2. Nerve injury

**Figure 2.**
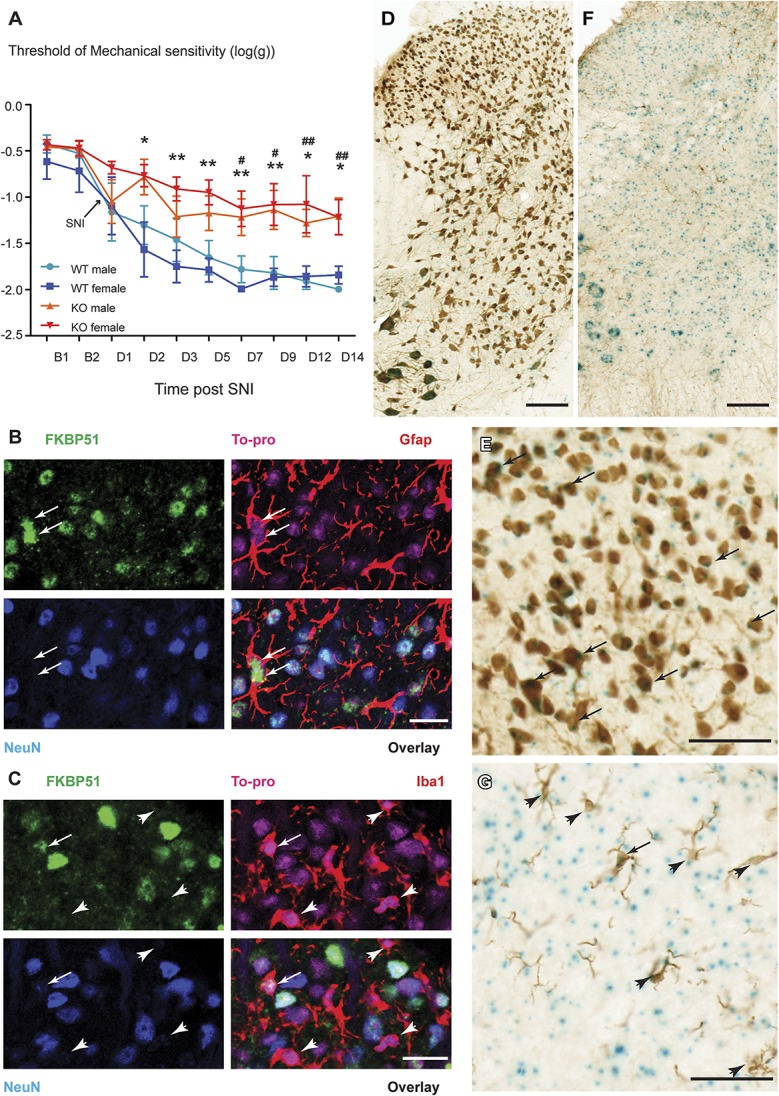
FKBP51 regulates neuropathic pain states across sexes and is often seen in neurones and sometimes in astrocytes in the superficial dorsal horn. (A) Mechanical sensitivity in female and male KO and WT mice after SNI (n = 6 per group) (2-way analysis of variance [ANOVA], TIME D1 to D14, factor “Genotype”: F_1,19_ = 39.6, *P* < 0.0001. Two-way ANOVA female only, TIME D1 to D14, factor “Genotype”: F_1,9_ = 21.4, *P* < 0.001. Two-way ANOVA male only, TIME D1 to D14, factor “Genotype”: F_1,10_ = 17.8, *P* < 0.01) *P<0.05, **P<0.001, ^#^*P*<0.05, ^#^^#^*P*<0.001. (B and C) FKBP51 immunoreactivity 7 days after SNI surgery. FKBP51 was often seen in neurones (stained with NeuN, blue, B and C), sometimes in astrocytes (stained with Gfap, red, B), and very rarely in microglia (stained with Iba1, red, C). (B and C) Cyan: To-pro, nuclear marker; scale bar: 15 μm. Arrows indicate astrocytes or microglia positive for FKBP51; arrowheads indicate microglia negative for FKBP51. (D–G) X-Gal stain confirming that FKBP51 (blue) is mainly expressed in neurones (brown, D and E) and very rarely in microglia (brown, F and G). Scale bar: 100 μm; arrows indicate neurones or microglia positive for X-gal; arrowheads indicate microglia negative for FKBP51. KO, knock-out; SNI, spared nerve injury; WT, wild-type.

We next investigated whether FKBP51 also regulated neuropathic pain states across sexes. Both KO male and KO female mice were less sensitive than their sex-matched WT littermates to neuropathic pain induced by SNI^11^ (Fig. 2A). There was no difference between females and males mechanical thresholds both at baseline and after surgery.

To explore the effects of the deletion of FKBP51 on GR signalling, we measured corticosterone levels in KO and WT mice before and after SNI, as we had performed previously in a model of ankle joint inflammation.^21^ We found that nerve injury induced an overall increase in plasma corticosterone levels across both genotypes (Table 1), but that plasma corticosterone levels were lower in KO mice both before and after nerve injury (Table 1), confirming our observation that *FKBP5* deletion leads to the suppression of corticosterone secretion both in naive mice and mice with persistent pain.^21^

**Table 1:**
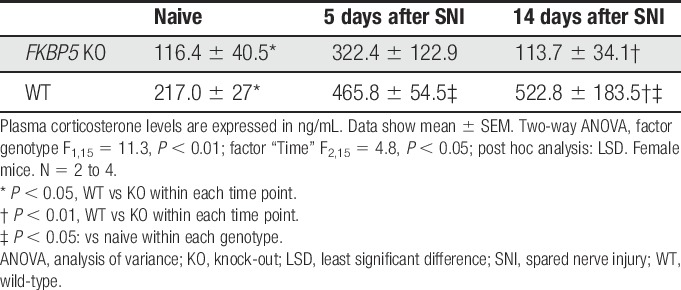
Corticosterone levels in *FKBP5* KO and WT mice before and after nerve injury.

We then wanted to characterise FKBP51 protein expression in mice with nerve injury, specifically in the superficial dorsal horn, where FKBP51 exerts its key role in the modulation of mechanical hypersensitivity.^21^ We had previously reported that FKBP51 was mainly expressed in neurons of the superficial dorsal horn of mice and rats that were naive or had an inflamed joint^15,21^ and this was supported by others.^6^ However, FKBP51 mRNA has also been found in cultured brain microglia.^42^ Because astrocytes and microglia are activated in the superficial dorsal horn after nerve injury, tissue from SNI animals was particularly suited to investigate the expression of FKBP51 in these cell types. Using a protocol of amplification with tyramide and an antibody from Santa Cruz that showed no specific stain in FKBP51 KO mice,^21^ we fully characterised the expression of FKBP51 in the superficial dorsal horn of mice. FKBP51 expression was mainly seen in neurons with 58% ± 4% of neurones expressing FKBP51 in naive mice and 44% ± 5% of astrocytes, or 6 FKBP51-positive neurones for every FKBP51-positive astrocyte (Fig. 2B). After nerve injury (7 days postsurgery), there was a significant increase in the proportion of neurones and astrocytes expressing FKBP51, with FKBP51 seen in 70% ± 5% of neurones on the injured side (F_1,4_ = 14.6, *P* < 0.05) and 60% ± 5% of astrocytes (F_1,4_ = 9.2, *P* < 0.05), or 7 FKBP51-positive neurones for every FKBP51-positive astrocyte. Microglia very rarely stained positive for FKBP51, with an average of 7% ± 5% of microglia positive for FKBP51, or 117 FKBP51-positive neurones for every FKBP51-positive microglia (Fig. 2C), and no increase after nerve injury. There was no difference in the number of neurones, astrocytes, and microglia expressing FKBP51 between male and female mice (data not shown). To confirm the low level of expression of FKBP51 in microglia, we used an X-Gal stain. The *FKBP5* KO mice contain a β-galactosidase reporter cassette which expresses wherever the *FKBP5* gene is normally expressed. Using this approach, we found that the gene *FKBP5* was expressed in 85% ± 1.5% of neurones and in 5% ± 1% of microglia in the dorsal horn of SNI mice (Figs. 2D–G).

Altogether, our data suggested that FKBP51 contributes to the development and maintenance of mechanical hypersensitivity after nerve injury across sexes, and although FKBP51 is mainly expressed in neurones, astrocytic mechanisms of FKBP51-driven nociceptive signalling cannot be excluded.

#### 3.1.3. A model of chemotherapy-induced pain

**Figure 3.**
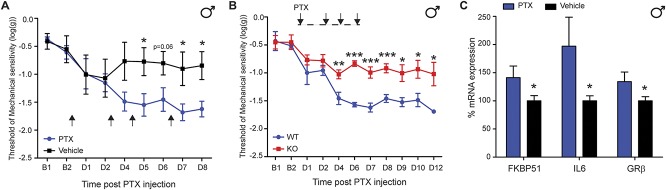
FKBP51 regulates the mechanical hypersensitivity seen in a model of paclitaxel-induced pain. (A) Mechanical sensitivity in wild-type (WT) mice after paclitaxel or vehicle treatment (n = 7/5) (2-way analysis of variance [ANOVA], TIME D4 to D8, factor “Treatment”: F_1,10_ = 6.6, *P* < 0.01). (B) Mechanical sensitivity in male KO and WT mice after paclitaxel treatment (n = 6/6) (2-way ANOVA, TIME D1 to D12, factor “Genotype”: F_1,10_ = 34.8, *P* < 0.0001). Data show mean ± SEM. *P < 0.05, **P < 0.01, ***P < 0.001, results of post hoc 1-way ANOVA. (C) RT-qPCR analysis of FKBP51, IL6, and GRβ in the dorsal horn 10 days after PTX injection (n = 5/5); data normalized to WT sham; *P < 0.05.

Here, we sought to investigate the involvement of FKBP51 in a pathological pain model induced by the chemotherapeutic drug paclitaxel. Paclitaxel treatment induced a significant decrease in mechanical sensitivity in adult mice (Fig. 3A) compared with vehicle. However, the effects observed in KO mice were significantly less severe than in WT mice (Fig. 3B), indicating that FKBP51 drives paclitaxel-associated mechanical hypersensitivity.

At the molecular level, we found that the paclitaxel-associated hypersensitivity was accompanied by an increase in FKBP51 expression in the superficial dorsal horn and an increase in GR_β_ expression and IL6 (Fig. 3C), as observed in inflammatory pain states (Fig. 1B)^21^ and after peripheral nerve injury.^44^

### 3.2. Pharmacological inhibition of FKBP51 significantly attenuates mechanical hypersensitivity in established long-term pain states

Finally, we characterised the effect of pharmacological blockade of FKBP51 using the only specific ligand available for in vivo use, the FKBP51 ligand SAFit2.^1,14,17^ We have previously shown that 1 single intrathecal administration of SAFit2 (4 μg in 2 μL) could reduce the hypersensitivity induced by ankle joint inflammation in male mice for 4 hours.^21^ Here, we explored the efficacy of various routes of administration of SAFit2 on established long-lasting pain states across sexes.

**Figure 4.**
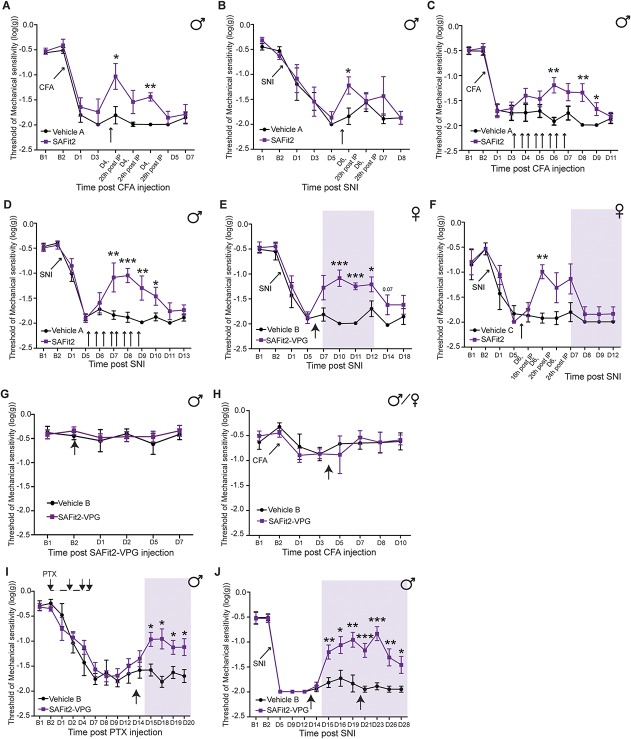
Pharmacological blockade of FKBP51 reduces the mechanical hypersensitivity seen in long-term pain states in both male and female mice. (A) Single subcutaneous injection of SAFit2 3 days after CFA in the ankle joint in WT males (n = 4/4) (2-way analysis of variance [ANOVA], TIME D3 to 28 h, factor “Treatment”: F_1,5_ = 21.6, *P* < 0.01). (B) Single subcutaneous injection of SAFit2 5 days after spared nerve injury (SNI) in males (n = 4/4) (2-way ANOVA, TIME D5 to D7, factor “Treatment”: F_1,6_ = 7.98, *P* < 0.05). (C) Repeated systemic i.p. injection of SAFit2 3 days after CFA in the ankle joint in males (n = 6/8) (2-way ANOVA, TIME D1 to D11, factor “Treatment”: F_1,12_ = 6.8, *P* < 0.05). (D) Repeated systemic i.p. injection of SAFit2 5 days after SNI in males (n = 6/8) (2-way ANOVA, TIME D1 to D13, factor “Treatment”: F_1,12_ = 8.8, *P* < 0.05). Intraperitoneal injections in (C and D): SAFit2 2 mg/mL in vehicle A. (E) Single subcutaneous injection of SAFit2-VPG (n = 5/5) in females (SAFit2 10 mg/mL, 10 mL/kg in vehicle B) 5 days after SNI (2-way ANOVA, TIME D7 to D13, factor “Treatment”: F_1,8_ = 17.1, *P* < 0.01). (F) Single subcutaneous injection of SAFit2 5 days after SNI in females (n = 4/4) (2-way ANOVA, TIME D5 to D7, factor “Treatment”: F_1,6_ = 6.0, *P* < 0.05) (SAFit2 10 mg/mL, 10 mL/kg in vehicle C). (G) Single subcutaneous injection of SAFit2-VPG in naive WT mice. (H) Single subcutaneous injection of SAFit2-VPG 3 days after CFA in the ankle joint in KO mice. (G and H) N = 3 per group. (I) Single subcutaneous injection of SAFit2-VPG (n = 6/6) in male after paclitaxel (SAFit2 10 mg/mL, 10 mL/kg in vehicle B) (2-way ANOVA, TIME D14 to D20, factor “Treatment”: F_1,10_ = 10.3, *P* < 0.01). Arrows indicate intraperitoneal injections: pointing-down arrows, paclitaxel injection; pointing-up arrow, SAFit2-VPG. (J) Repeated subcutaneous injection of SAFit2-VPG (n = 8/8) in males 12 days after SNI (SAFit2 10 mg/mL, 10 mL/kg in vehicle B). SAFit2-VPG was injected on D12 and D19; (2-way ANOVA, TIME D15 to D26, factor “Treatment”: F_1,6_ = 48.3, *P* < 0.001). (A–J) B: baseline; D: day. (E, F, I and J): the purple band highlights a period of 5 days starting 48 hours after SAFit2 injection. Data show mean ± SEM. *P < 0.05, **P < 0.01, ***P < 0.001, results of post hoc 1-way ANOVA. SAFit2 or vehicle injection time is indicated by vertical arrows (small arrow for SAFit2 and big arrow for SAFit2-VPG). CFA, complete Freud adjuvant; i.p., intraperitoneal; KO, knock-out; VPG, vesicular phospholipid gel; WT, wild-type.

A single i.p. injection of SAFit2 (20 mg/kg) was able to reduce injury-induced mechanical hypersensitivity when injected when the pain state was fully developed 3 days after CFA injection in the ankle joint (Fig. 4A) or 5 days after SNI surgery (Fig. 4B). As expected, the effect of a single injection of SAFit2 lasted no more than 8 hours in both models, and we decided to test the effect of repeated injections of SAFit2 (20 mg/kg), twice daily, over 4 days. After this protocol, SAFit2 significantly reduced the injury-induced hypersensitivity for up to 4 days in the joint inflammatory and neuropathic models (Figs. 4C and D).

To reduce animal stress and improve the efficacy of the drug treatment, we explored a state-of-the art method of sustained release of FKBP51 using VPGs.^1^ Here, SAFit2-VPG was injected subcutaneously 5 days (Fig. 4E) after SNI surgery (SAFit2 100 mg/kg). A single injection of SAFit2-VPG reduced the SNI-induced mechanical hypersensitivity for at least 7 days. Comparing the subcutaneous unretarded delivery of an SAFit2 solution containing the same dose of SAFit2 (SAFit2 100 mg/kg) (Fig. 4F), we could confirm the depot effect of the VPG and the slow release of SAFit2 from the liposome matrix by diffusion, leading to a significantly sustained pharmacological effect of SAFit2. SAFit2-VPG had no effect in naive mice (Fig. 4G), confirming that FKBP51 did not regulate naive mechanical threshold nor in CFA-injected *FKBP5* KO mice (Fig. 4H), confirming the specificity of the ligand SAFit2.

We then tested if SAFit2 could improve the mechanical hypersensitivity that develops after PTX treatment. Mice received a single injection of SAFit2-VPG (SAFit2 100 mg/kg) 12 days after the first injection of PTX, when the mechanical hypersensitivity was fully established. As expected, SAFit2-VPG significantly reduced the mechanical hypersensitivity for at least 5 days (Fig. 4I).

Finally, we investigated whether delayed administration of SAFit2-VPG could improve long-term pain states that had been fully established for at least 1 week and whether repeated administration of SAFit2-VPG would maintain the improvement of the pain state. When given 12 days after nerve injury, SAFit2-VPG (SAFit2 100 mg/kg) once again reduced the SNI-induced mechanical hypersensitivity and when the injection was repeated 7 days after the first one, the reduced hypersensitivity was maintained for at least 16 days (Fig. 4J). Altogether, our data strongly suggest that FKBP51 is a promising pharmacological target for the treatment of persistent pain states across sexes.

## 4. Discussion

In this study, we have demonstrated that FKBP51 was the driver of long-term pain states of varied aetiology but all associated with the upregulation of IL6 at spinal cord level. This regulation of persistent pain states by FKBP51 was seen across sexes and occurred through modulation of glucocorticoid signalling. Then, using the unique FKBP51 ligand for in vivo use, SAFit2, encapsulated in a state-of-the-art VPG for slow release, we have shown that pharmacological inhibition of FKBP51 reduced the established mechanical hypersensitivity induced by joint inflammation, nerve injury, and the anticancer drug paclitaxel. We therefore propose that FKBP51 is a new pharmacologically tractable target suitable for the treatment of persistent pain in both males and females.

Here, we demonstrated that FKBP51 drives long-term pain states across sexes and established that a pharmacotherapy targeting FKBP51 would alleviate long-lasting pain states in males and females. This was a very important translational step forward because the mechanisms of development of chronic pain states differ between sexes and sex differences in analgesic response to pain treatment are often seen.^2,32^ Our data, however, suggest that pharmacological inhibition of FKBP51 is sufficient to improve the mechanical sensitivity that develops in a variety of long-term pain states in both female and male mice. It is currently understood that sex differences observed in chronic pain states arise from differences in immune system between males and females. Immune cells are known to contribute to the development of chronic pain, and microglial–neurones interactions, in particular, have been believed to be crucial to the establishment of long-lasting pain states.^3,37,41^ However, although males and females show similar microglial activation in the superficial dorsal horn after injury, females are not reliant on this activation for the development of mechanical hypersensitivity while males are.^31,34^ Our observation that FKBP51 is mainly expressed in neurones and hardly ever seen in microglia in the spinal cord would argue that the mechanisms of regulation of persistent pain states by FKBP51 are independent of immune cells–related events that often precede neuronal changes at spinal cord levels in chronic pain states.

At the molecular level, we found that the persistent pain states that were driven by FKBP51 were associated with (1) changes in glucocorticoid signalling, as indicated by the increased expression at spinal cord level of GR_β_, the GR isoform associated with glucocorticoid resistance^20,28^ and increased levels of plasma corticosterone, and (2) an upregulation of IL6 expression in the dorsal horn, as shown by our data and work from others recently reviewed by Zhou et al.^44^ Although the requirement for intact GR signalling for the full development of persistent pain states has already been established,^33,39,40^ the downstream events that drive these pain states are not fully understood. The *N*-methyl-d-aspartate (NMDA) receptor was found upregulated in the spinal cord after chronic constriction nerve injury and had been suggested as a crucial link between GR signalling and persistent neuropathic pain induced by chronic constriction nerve injury.^40^ However, we found no changes in expression of the NMDA receptor after inflammation of the ankle joint (data not shown) at a time point where FKBP51 was upregulated and mice hypersensitive. These results suggested that the NMDA receptor was unlikely to be central to the mechanisms of maintenance of persistent pain by FKBP51. However, our findings that IL6 was upregulated in the 3 pain models we have studied suggest that IL6 could be a crucial link. Administration of IL6 can cause mechanical allodynia and intrathecal injection of anti-IL6 neutralizing antibody can alleviate pain-related behaviours,^24,44^ indicating a critical role for IL6 in the maintenance of persistent pain states. Because GR can directly regulate IL6 transcription by complex binding to IL6 promoter regulatory elements^38^ and because the GR agonist dexamethasone can induce IL6 expression in rodent neurons,^27^ it is possible that the regulation of chronic pain states by FKBP51 occurs through IL6, downstream of glucocorticoid signalling. The fact that FKBP51 and IL6 are mainly expressed in neurones in the superficial dorsal horn would support this idea. Importantly, injured *FKBP5* KO mice had lower levels of IL6 in the superficial dorsal horn after CFA injection in the ankle joint^21^ and after nerve injury (data not shown). Moreover, IL6 is not upregulated in the dorsal horn in short-term pain states that are not modulated by FKBP51, such as the inflammatory pain state initiated by IL6 injection in the hind paw.^21^ Although the regulation of IL6 expression in the dorsal horn by glucocorticoid signalling remains to be demonstrated, our data certainly provide further support for the regulation of persistent pain states by GR signalling at spinal cord level.

Here, we were able to replicate the effect of FKBP51 deletion on injury-induced mechanical hypersensitivity using the FKBP51 ligand SAFit2. Using the same doses of SAFit2 in vivo (20 mg/kg or 100 mg/kg for SAFit2-VPG), others were also able to replicate the anxiolytic properties, FKBP51 deletion^17^ and its effects on body weight regulation and glucose tolerance.^1^ This study also showed that high and stable SAFit2 plasma levels can be achieved in vivo, and that the slow release SAFit2-VPG formulation reaches plasma SAFit2 concentrations comparable with the repeated-twice daily 20 mg/kg injection.^1^ Importantly, SAFit2 has a selectivity for FKBP51 over FKBP52 >10,000^14^ and a >500× selectivity windows for a panel of 43 behaviourally relevant central nervous system receptors (https://pdspdb.unc.edu/pdspWeb; unpublished data Dr Felix Hausch). Although the FKBP51 functions modulated by SAFit2 currently remain unclear, our data could suggest that SAFit2 modulates FKBP51 regulation of GR sensitivity. Whether SAFit2 can reverse the increase in IL6 observed in our animal models also remains to be investigated.

Crucially, we were able to offer long-lasting pain relief in persistent pain states, in both male and female mice, using a single injection of SAFit2 encapsulated in a VPG allowing the slow release of SAFit2.^1^ Vesicular phospholipid gels are highly concentrated, semisolid dispersions of phospholipids in aqueous medium^8^ that display excellent biocompatibility because of their composition of solely phospholipids and water or buffer^9^ and can provide release rates over several weeks. Vesicular phospholipid gels have been used in the past as depot formulations for the sustained release of pharmaceutical drugs, including peptides and proteins.^9,16,36^ The capability of VPGs to encapsulate water-soluble drugs, as well as drugs with poor solubility in aqueous medium, makes them perfectly suited as a sustained release system for a substance such as SAFit2. Hydrophobic interactions between the relatively lipophilic SAFit2 and the phospholipids of the formulation lead to binding and incorporation of the drug in the hydrophobic areas of the phospholipid bilayers of the liposome matrix, and SAFit2 has to diffuse through several densely packed hydrophilic and lipophilic compartments on its way out of the depot, resulting in a strong retardation. Here, SAFit2-VPG was injected subcutaneously, once the pain states were fully established, and was able to reduce the mechanical hypersensitivity for at least 7 days after a single injection, and at least 14 days when reinjected after 7 days, indicating that chronic inhibition of FKBP51 could offer long-lasting pain relief.

SAFit2 resolved mechanical hypersensitivity when administrated as late as 12 days after nerve injury, suggesting that inhibition of FKBP51 could be a promising treatment for chronic pain for patients with late diagnosis. We have also shown that FKBP51 could be targeted to reduce the hypersensitivity seen in a murine model of chemotherapy-induced peripheral neuropathy, even when given once the pain state was fully established. Chemotherapy-induced peripheral neuropathy is a common debilitating side effect of anticancer drugs and unfortunately, with no treatment options for chemotherapy-induced peripheral neuropathy, clinicians often need to reduce the dose of the chemotherapeutic agent or discontinue its use,^30^ which leads to poorer survival rates. Our data suggest that FKBP51 inhibition could provide pain relief to cancer patients treated with paclitaxel and therefore offer hope for prolonged anticancer treatments.

## 5. Conclusions

In conclusion, we have demonstrated that FKBP51 is an exciting new pharmacological target for the treatment of chronic pain states across sexes. Inhibition of FKBP51 with the state-of-the-art ligand SAFit2 reduced the hypersensitivity that develops after ankle joint inflammation, nerve injury, and paclitaxel treatment. These long-term pain states have distinct underlying mechanisms, suggesting that FKBP51 could be targeted for the treatment of a large number of persistent pain states across sexes. Moreover, when encapsulated in VPG, a single injection of SAFit2 led to a long-lasting relief of hypersensitivity indicating a strong translational potential for chronic treatment by FKBP51 inhibition. Importantly, individuals with variants in the *FKBP5* gene have altered pain sensitivity after trauma,^7^ lending confidence that pharmacological inhibition of FKBP51 could indeed help patients suffering with chronic pain.

## Conflict of interest statement

The authors have no conflict of interest to declare.

This work was supported by the United Kingdom Medical Research Council grant G1100577, an NIH Fast Track Grant and a grant from the Pain Relief Foundation to S.M. Géranton. T. Mao was supported by the Chinese Scholarship Council and F. Hausch received funding from the StMWIVT (BIO-1601-0003) and ERA-IB7 (031B0269B). The authors have declared that no conflict of interest exists.

## Supplementary Material

SUPPLEMENTARY MATERIAL

## Appendix A. Supplemental digital content

Supplemental digital content associated with this article can be found online at http://links.lww.com/PAIN/A550.
